# Photodynamic Therapy (PDT) in Prosthodontics: Disinfection of Human Teeth Exposed to *Streptococcus mutans* and the Effect on the Adhesion of Full Ceramic Veneers, Crowns, and Inlays: An In Vitro Study

**DOI:** 10.3390/biomedicines10010144

**Published:** 2022-01-10

**Authors:** Corina Elena Tisler, Radu Chifor, Mindra Eugenia Badea, Marioara Moldovan, Doina Prodan, Rahela Carpa, Stanca Cuc, Ioana Chifor, Alexandru Florin Badea

**Affiliations:** 1 Department of Prosthetic Dentistry and Dental Materials, Iuliu Hatieganu University of Medicine and Pharmacy, 32 Clinicilor Street, 400006 Cluj-Napoca, Romania; tisler.corina@umfcluj.ro; 2 Department of Preventive Dental Medicine, Iuliu Hatieganu University of Medicine and Pharmacy, Avram Iancu 31, 400083 Cluj-Napoca, Romania; mebadea@umfcluj.ro (M.E.B.); ioana.chifor@umfcluj.ro (I.C.); 3 Department of Polymer Composites, Institute of Chemistry “Raluca Ripan”, University Babes-Bolyai, 400294 Cluj-Napoca, Romania; marioara.moldovan@ubbcluj.ro (M.M.); doina.prodan@ubbcluj.ro (D.P.); 4 Department of Molecular Biology and Biotechnology, Faculty of Biology and Geology, Babeș Bolyai University, 1 M. Kogălniceanu Street, 400084 Cluj-Napoca, Romania; rahela.carpa@ubbcluj.ro; 5 Department of Morphological Sciences, Discipline of Anatomy and Embryology, Faculty of General Medicine, Iuliu Hatieganu University of Medicine and Pharmacy, 3–5 Clinicilor Street, 400006 Cluj-Napoca, Romania; alexandru.badea@umfcluj.ro

**Keywords:** photodynamic therapy, biofilm, ceramic, adhesion, pull-out test, SEM

## Abstract

The use of PDT in prosthodontics as a disinfection protocol can eradicate bacteria from tooth surfaces by causing the death of the microorganisms to which the photosensitizer binds, absorbing the energy of laser light during irradiation. The aim of the study was to investigate the capacity of PDT to increase the bond strength of full ceramic restorations. In this study, 45 extracted human teeth were prepared for veneers, crowns, and inlays and contaminated with *Streptococcus mutans*. Tooth surfaces decontamination was performed using a diode laser and methylene blue as a photosensitizer. The disinfection effect and the impact on tensile bond strength were evaluated by scanning electron microscopy (SEM) and pull-out tests of the cemented ceramic prosthesis. Results show that the number of bacteria was reduced from colonized prepared tooth surfaces, and the bond strength was increased when PDT was used. In conclusion, the present study indicates that using PDT as a protocol before the final adhesive cementation of ceramic restorations could be a promising approach, with outstanding advantages over conventional methods.

## 1. Introduction

In recent years, dentistry has experienced a wide development, and patient demands are aligned with the present restorative possibilities. All-ceramic restorations can regain lost functions, combining the strength of materials with a clearly superior and stable aesthetic over time, while adhesive cements ensure adequate bonding. Furthermore, these materials require minimum invasive preparations, as they are able to preserve and prolong teeth integrity.

Cementation in fixed prosthodontics as a final clinical step can be very challenging. The longevity of the restorations depends on the accuracy of the two main procedures that are performed in the last visit to the clinic—disinfection of the prepared tooth and cementation.

Photodynamic therapy is a non-invasive approach that involves a photosensitizer, a visible light of an appropriate wavelength, and the production of reactive oxygen species (mainly singlet oxygen), which immediately causes phototoxicity and leads to serious bacterial damage and death [1,2,3,4,5]. Its efficacy over *Streptococcus mutans* (a Gram-positive bacteria and the main responsible for dental caries) has been demonstrated, both in the stage of free microorganism and while organized in biofilms [4,5,6,7]. PDT is able to strongly reduce the number of bacteria from colonized dental surfaces.

The positive effect of photodynamic therapy can also extend to adhesion when used as a decontamination protocol before cementation. We did not find any studies able to reveal the impact of PDT on prosthodontic cementation, while in endodontics and orthodontics, it had a negative impact or was effectless on the bond strength and mechanical properties of dentin from the intracanal prosthetic space [8,9,10,11] and bond strength of orthodontic brackets [12].

Over the past several years, dental ceramics used in prosthodontics have been upgraded, achieving a great improvement of their mechanical properties due to the actual processing techniques and enhanced microstructures [13]. Lithium disilicate—the most representative material of glass ceramics—is a biocompatible and esthetic material with remarkable mechanical properties and a wide applicability, used for inlays, veneers, and crowns (anterior and posterior) fabrication [14]. These prostheses represent single-tooth restorations generally made of ceramic. Their major advantage is they require minimal preparations and allow dental tissues conservation. The highlighted advantages of inlays, veneers, and crowns are completed by adhesive cementation.

The adhesion of all-ceramic restorations has multiple advantages, such as higher retention and improved marginal adaptation. Resin-based adhesive cements are widely used for inlays, crowns, and veneers cementation, especially the dual-cured type, which gives a better polymerization control and an increased working time [15].

The substrate to which ceramic is bonded has great importance, as adhesion to the enamel is superior to the one that can be achieved onto dentin. In consequence, dental preparations limited to enamel have a higher bond strength than the ones at a depth that implies both enamel and dentin. For this reason, the bonding of ceramic veneers only to dentin should be avoided or performed with great caution [16].

Nevertheless, the examination of the tensile strength of luting cements can be performed by using a pull-out test to achieve axial oriented forces that lead to dislodgement of restorations cemented to extracted human teeth [17].

The novelty of the present research lies in the adhesion testing of three types of lithium disilicate prostheses before and after PDT, along with the antibacterial effect when an atypical protocol was used.

The aim of this study is to evaluate the efficiency of photodynamic therapy in the field of prosthodontics by simulating a clinical cementation protocol of all-ceramic restorations (inlays, veneers, and crowns) and to underline the double advantage that a single operation can have—simultaneous disinfection and bond strength enhancement. Our main purpose is to analyze the capacity of PDT of interfering with the adhesion of lithium disilicate ceramic prostheses by mechanically testing the bonded final restorations. The null hypothesis was that PDT had no effect on disinfecting prepared teeth or increasing the adhesion to all-ceramic restorations.

## 2. Materials and Methods

### 2.1. Prosthodontic Preparation

The samples were represented by 45 extracted human teeth (incisors, premolars, and molars) that were divided into 3 groups as follows: 15 teeth were prepared for veneers (with an extension of the preparation on the oral surface; noted from 1F to 15F), 15 for class I inlays (noted from 1I to 15I), and 15 for crowns (noted from 1C to 15C). All preparations were made using diamond burs fixed into a high-speed, high-torque handpiece (electric motor) at depths that afford the placement of ceramic restorations, using an identical preparation and finishing protocol. Due to the difficulties encountered during the collection, handling, and storage of real human saliva, artificial saliva was preferred as a storage material for teeth before and after prosthodontic preparation. Artificial saliva formulations were developed at the Department of Polymer Composites, Institute of Chemistry “Raluca Ripan” and contain Na_2_HPO_4_, NaHCO_3_, CaCl_2_, H_2_O, and HCl.

### 2.2. Bacterial Contamination

The strain used was *Streptococcus mutans* ATCC 25,175 from the collection of the Laboratory of Microbiology, Faculty of Biology and Geology, UBB, Cluj.

To obtain the bacterial suspension, the BHI-T culture medium was inoculated with the *Streptococcus mutans* strain which was then incubated for 24 h at 37 °C. After the growing period, the bacterial suspension was used as the inoculum for the 45 tubes with BHI-T culture medium. A volume of 500 µL of 0.5 MacFarland bacterial suspension was inoculated into tubes with a BHI-T medium. Then, aseptically, one tooth was placed in each tube.

The tubes with teeth placed in the BHI-T culture medium and inoculated with the bacterium under study were incubated for 8 days at a temperature of 37 °C. Immediately after the incubation period, teeth were removed from the immersion environments and placed on aluminum stubs with the prepared surface facing upwards. The specimens were then examined using SEM at a low vacuum, at a pressure of 80 Pa, and with an acceleration voltage of 30 kV. Scanning electron microscopy (SEM-Inspect S, FEI) examination was performed to identify the presence of bacterial biofilm on prepared tooth surfaces at a magnification of ×5000.

### 2.3. Photodynamic Therapy Protocol

PDT was performed using the SiroLaser Blue (Dentsply Sirona, New York, NY, USA) from the Department of Preventive Dental Medicine, Iuliu Hatieganu University of Medicine and Pharmacy, Cluj. Methylene blue (MB) was used as a photosensitizer and washed using a gradated syringe with Kaqun water (oxygen-rich, alkaline water).

For every group of teeth from 1 to 10 MB gel 1% was applied on the prepared surfaces for 3 min and then washed under an easy jet of 3 mL Kaqun water for 20 s. The MultiTip of 8 mm was chosen as the irradiation laser tip. The diode SiroLaser Blue with 660 nm wavelength was set at 100 mW power in the continuous mode and applied for 180 s. This protocol was repeated identically for every tooth from 1 to 10, while teeth from 11 to 15 from every group were washed only with 3 mL of Kaqun water for 20 s, without PS or laser irradiation. SEM examination was repeated to observe bacterial presence on teeth subjected to PDT.

### 2.4. Fabrication and Cementation of Prosthodontic Restorations (Veneers, Inlays, and Crowns)

Impressions of all teeth were taken with Variotime (Heraeus, Hanau, Germany) polyvinyl siloxane using the sandwich impression technique. Gypsum casts were poured, on which wax patterns were then modeled. After modeling, a prefabricated wax rod was placed on each wax model, parallel with the path of insertion. For the fabrication of the prosthesis, IPS e.max PRESS ceramic ingots were used. The selected color was A2 from the Vita Classical Shade Guide. Ceramic pressing was performed with Programat EP 3010 oven. Final restorations were not glazed, and the resulting ceramic rods were kept as a structure of the restorations.

After conducting the previously described PDT, cementation was performed for every tooth, according to the instructions of the manufacturer of Variolink Esthetic dual-cure (DC) luting composite. Restorations were placed on teeth using Variolink Esthetic try-in paste, and the adaptation was checked. Monobond Etch & Prime (self-etching glass-ceramic primer) was applied with a micro brush on the internal surfaces of restorations for 60 s and then spread with a strong stream of air. Prepared surfaces were etched using orthophosphoric acid gel 37%, for 15–30 s on enamel, and 10–15 s on dentin. The etching agent was rinsed thoroughly with a stream of water. Teeth surfaces were dried until the etched enamel appeared chalky white. Starting with the enamel, tooth surfaces were coated with Adhese Universal for 20 s and then dispersed with oil- and moisture-free compressed air. Light curing was performed for 10 s using Demi Plus Kerr Dental Curing Light. Variolink Esthetic DC was applied with an application tip directly to the internal surface of the restoration (for veneers and crowns) and in the cavity (for inlays). The ceramic prosthesis was then seated and held in place during excess removal. Excess material was light cured with a polymerization light for 2 s at 10–15 mm by running the light probe along the entire cement line and removed immediately with a scaler. Restoration margins were covered with liquid strips immediately after excess removal to prevent oxygen inhibition and light cured for 10 s. Margins and cement lines were polished with Kenda polishers. SEM examination was performed to explore the bond interface at the tooth–prosthesis junction.

### 2.5. Pull-Out Test

Each tooth was incorporated in self-curing acrylate at both ends—the ceramic rod and the root—leaving the crown free of acrylate. After the material setting, the whole assembly was kept in artificial saliva for 24 h.

**Figure 1:**
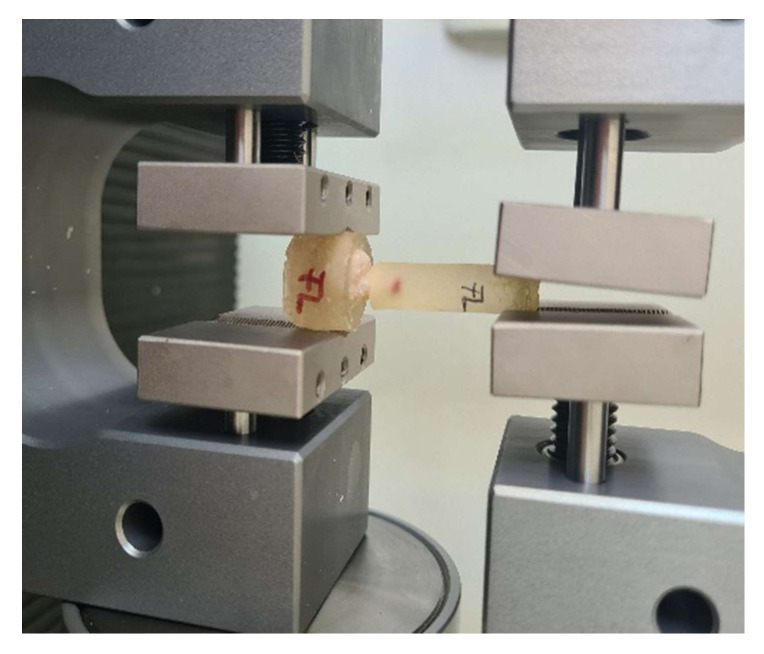
Pull-out mechanical test using LLOYD LR5k Plus.

Pull out-test was performed (Figure 1) by using a Lloyd LR5k Plus dual-column mechanical testing machine (Ametek/Lloyd Instruments, Germany, provided with a cell with a maximum recording force of 5KN), at a crosshead speed of 1 mm/min (ASTM D638 standard), and the data were processed using NexygenPlus software. For each investigated group, 15 evaluations (10 teeth with PDT and 5 teeth without PDT) were performed. Measurements that had a difference of ±15% of the measured average value were eliminated. The results were subjected to ANOVA one-way statistical analysis (α-0.05) and Tukey’s ad hoc test using the Origin 2019b Graphing and Analysis (Origin Lab) software (Northampton, MA, USA). Fracture areas were evaluated by SEM and optical microscope (Zeiss Stemi 2000-C Stereo Microscope 6.5x–50x, Germany).

## 3. Results

### 3.1. Effectiveness of Disinfection through Photodynamic Therapy

**Figure 2:**
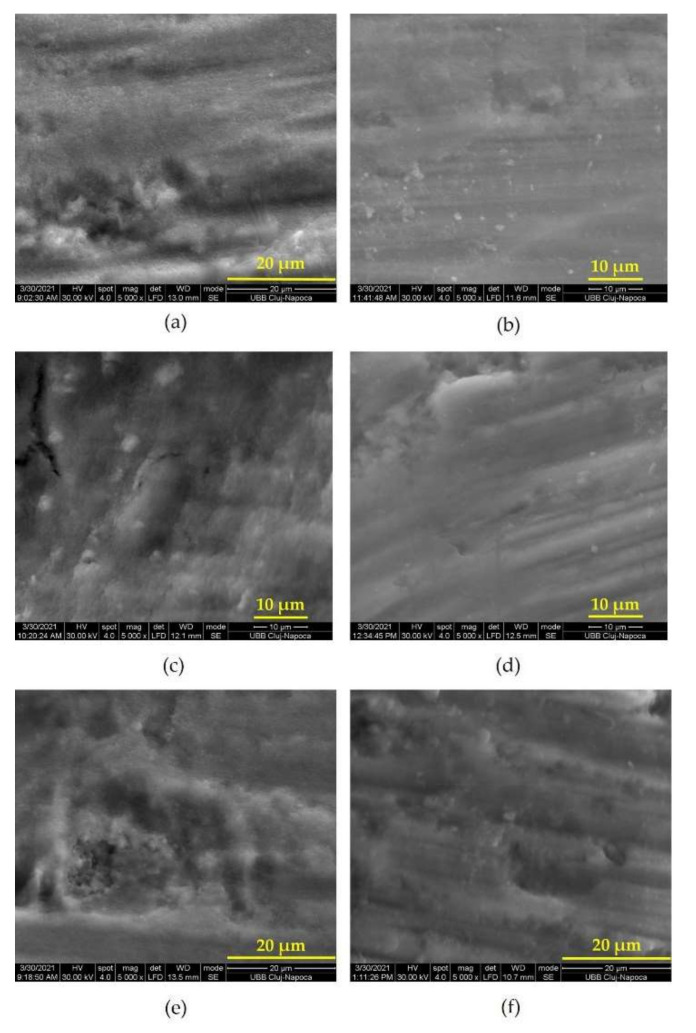
SEM images of contaminated teeth surface before and after PDT: (**a**) expansive colonization by *S. mutans*, biofilm covering entire veneer preparation; (**b**) SEM view after PDT on veneer preparation surface; (**c**) *S. mutans* biofilm on crown preparation; (**d**) crown preparation surface after PDT; (**e**) SEM image of *S. mutans* biofilm on inlay preparation; (**f**) inlay preparation surface after PDT.

This study assessed the effectiveness of using PDT as a method of disinfection of prepared coronary surfaces of extracted teeth. SEM analysis of contaminated prepared dental surfaces showed the appearance of *Streptococcus mutans* biofilm consisting of an agglomeration of cocci that completely covered the dental structures (Figure 2a,c,e). The 8-day-old biofilm revealed cells with a particular arrangement—such as solitary cells positioned next to each other—which had lost their specific chain conformation.

The antibacterial effect on photodynamic therapy was highlighted by post-irradiation images that showed the disappearance of the bacterial biofilm and the presence of few solitary remaining bacteria (Figure 2b,d,f). PDT managed to reduce the number of colonizing streptococci on the surface of dental preparations.

### 3.2. Appearance of Tooth–Prosthesis Interface after Adhesive Cementation

**Figure 3:**
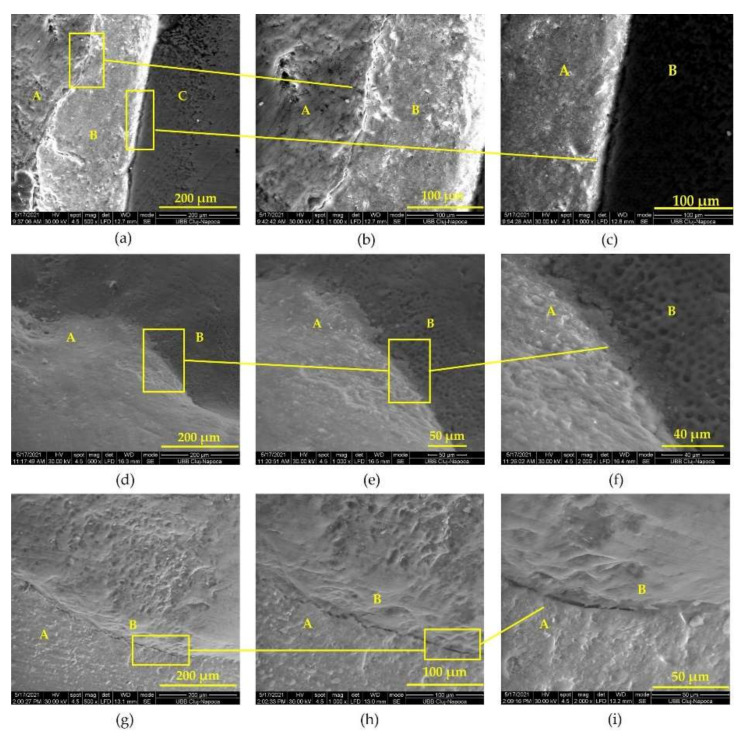
SEM representation of adhesion: (**a**) SEM image of the IPS e.max PRESS veneer interface area (A), adhesive cement (B), enamel area (C); (**b**) SEM image of the IPS e.max PRESS veneer (A), cement (B) interface; (**c**) SEM image of the interface adhesive cement (B), tooth enamel (C); (**d**) SEM view of ceramic inlay (A), tooth dentin (B), interface ×500; (**e**) SEM view of ceramic inlay (A), tooth dentin (B), interface ×1000; (**f**) SEM view of ceramic inlay (A), tooth dentin (B), interface ×2000; (**g**) SEM view of tooth enamel (A), ceramic crown (B), interface ×500; (**h**) SEM view of tooth enamel (A), ceramic crown (B), interface ×1000; (**i**) SEM view of tooth enamel (A), ceramic crown (B), interface ×2000.

SEM images of the adhesion between IPS e.max PRESS ceramic veneers, inlays, and crowns highlight qualitative cementation with an appropriate marginal adaptation to tooth structures (Figure 3).

### 3.3. Adhesion Pull-Out Test

**Figure 4:**
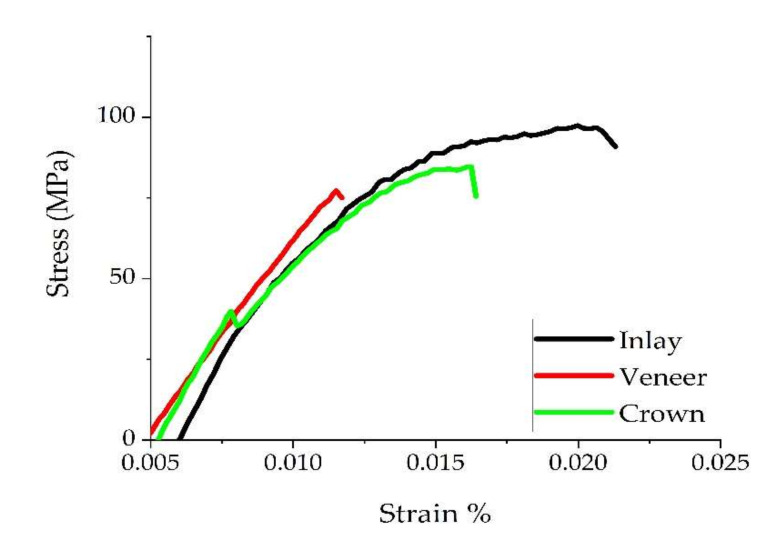
Comparison of the tensile strength highest values from each examined group when PDT was used.

**Figure 5:**
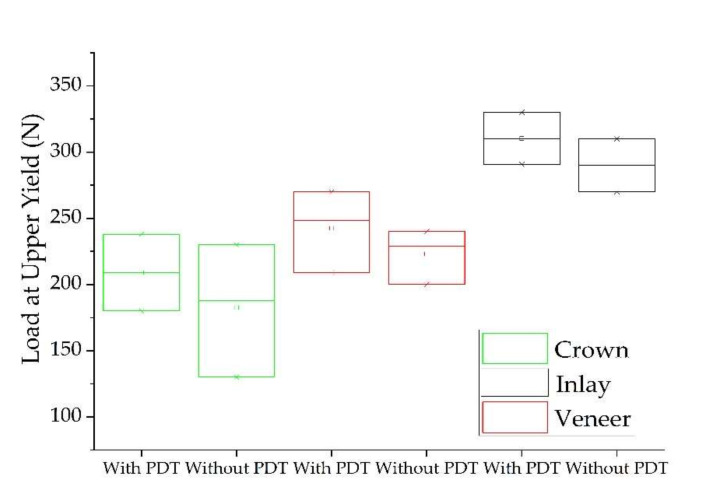
Comparison of load at upper yield between examined groups with and without PDT.

The results of the pull-out test for the examined groups (crown, veneer, and inlay) are summarized in Table 1. The highest values of tensile strength of specimens treated with PDT (Figure 4) and comparison of load at upper yield between groups (Figure 5) are graphically represented. Based on the comparison of Anova one-way test results between the groups of teeth with and without photodynamic therapy, there were no different statistical semificatives between them for any of the groups of materials (*p* ≥ 0.05). Based on the comparison of the values between all three groups (with and without photodynamic therapy) of restorations, there were different values of *p*, which did not show large statistically significant differences. In Tukey’s test, groups with the same letter are without statistically significant differences (tensile strength), and those with different notations reflect significant differences between them (crown and veneer with inlay for load at maximum load and load at upper yield).

**Figure 6:**
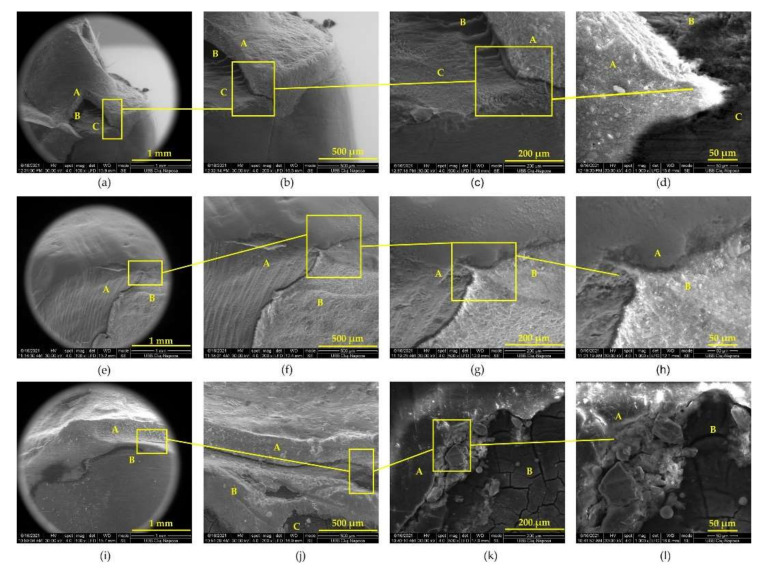
SEM examination of fracture areas after mechanical pull-out test: (**a**) SEM image of the veneer fracture zone: ceramic material (A), residual cement (B), tooth dentin (C) ×100; (**b**) SEM image of the veneer fracture zone: ceramic material (A), residual cement (B), tooth dentin (C) ×200; (**c**) SEM image of the veneer fracture zone: ceramic material (A), residual adhesive cement (B), tooth dentin (C) ×500; (**d**) SEM image of the veneer fracture zone: ceramic material (A), residual adhesive cement (B), tooth dentin (C) ×1000; (**e**) SEM view of the inlay cavity after debonding: prepared marginal dentin (A), residual adhesive cement (B) ×100; (**f**) SEM view of the inlay cavity after debonding: prepared marginal dentin (A), residual adhesive cement (B) ×200; (**g**) SEM view of the inlay cavity after debonding: prepared marginal dentin (A), residual adhesive cement (B) ×500; (**h**) SEM view of the inlay cavity after debonding: prepared marginal dentin (A), residual adhesive cement (B) ×1000; (**i**) SEM view of the fractured ceramic crown (A), tooth dentin (B) ×100; (**j**) SEM view of the fractured ceramic crown (A), residual adhesive cement (B), tooth dentin (C) ×200; (**k**) SEM view of the fractured ceramic crown (A), tooth dentin (B) ×500; (**l**) SEM view of the fractured ceramic crown (A), tooth dentin (B) ×1000.

**Figure 7:**
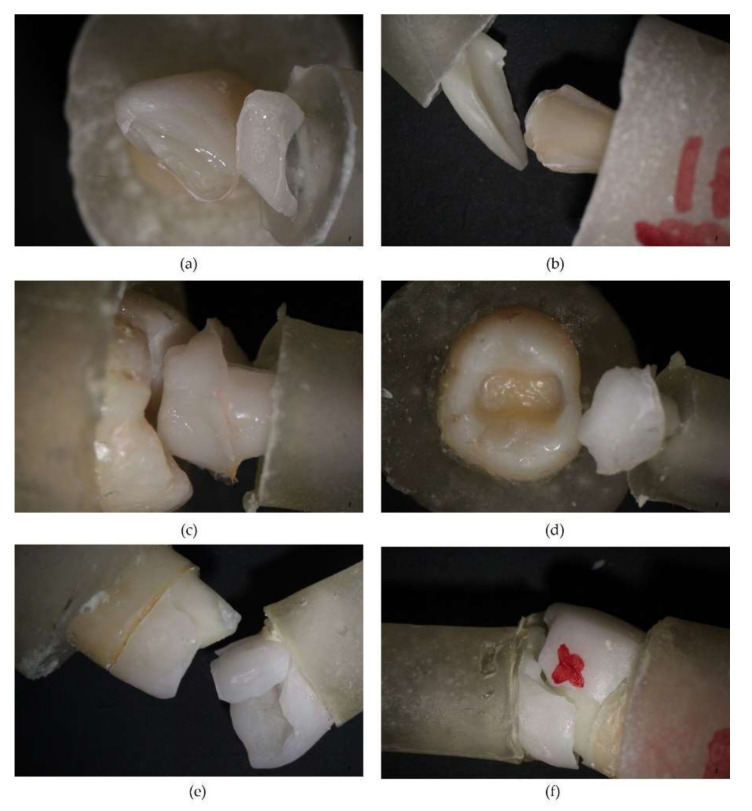
Optical microscope evaluation after mechanical pull-out test: (**a**) veneer fractured specimen; (**b**) fractured ceramic veneer after decementation; (**c**) inlay debonded specimen; (**d**) inlay restoration and preparation after debonding; (**e**) fractured crown restoration; (**f**) aspect of ceramic crown after decementation.

Fracture areas were highlighted by scanning electron microscopy (Figure 6) for veneer and crown groups, and prepared dentin was analyzed for debonded inlay group. SEM views revealed cracked subjacent dentin for veneers and crowns, fractured pieces of IPS e.max PRESS, and remaining Variolink Esthetic DC onto the dental surface alternating with areas where it was missing. For the inlay group, the specific detail was the presence of adhesive cement on the tooth, suggesting that fractures occurred at the cement–ceramic inlay interface. Optical microscopy images revealed veneers and crown specimens failure by fracture and inlay specimens failure by debonding (Figure 7).

## 4. Discussion

Given the increased applicability of photodynamic therapy in dentistry, our goal was to explore a less addressed branch. We studied the use of this therapy in fixed prosthetics as an alternative to conventional procedures for disinfecting previously prepared dental abutments and explored possible effects on the immediate adhesive cementation of ceramic prostheses frequently performed by dentists in their current practice. Due to changes in the evaluated properties when PDT was applied, the null hypothesis was rejected.

For bacterial inoculation, we selected the *Streptococcus mutans* strain, given that it is mainly responsible for the appearance of caries processes. An essential feature of this bacterium is that it has receptors that enable it to adhere to the hard dental tissues [18]. The carious potential of this bacterium is accentuated by the ability to secrete glucan (which promotes bacterial adhesion and biofilm formation) and lactic acid, also by its acid tolerance mechanism [19]. Previous studies evaluated PDT’s efficacy on *S. mutans* biofilms incubated for 5 days [4,7], 3 days [20], 48 h [21], respectively, while others [22] used a 7-day old biofilm. Our streptococcal biofilm also visibly developed on prepared teeth after 8 days. We chose this specific period considering that usually, a prosthetic restoration is finalized in about a week, during which the tooth is directly exposed to the oral bacterial flora if no provisional restorations are used or if they fracture.

Scanning electron microscopy is a common method of examining surface details by generating a three-dimensional image. This technique allows a direct examination performed at a high resolution [7,23]. In the present study, we verified the biofilm formation on the prepared areas of teeth. SEM images highlighted the presence of agglomerated cocci and poorly represented extracellular matrix structures, in accordance with other *S.mutans* biofilm exposures [7,22].

Although it was reported that the use of photodynamic therapy in fixed prosthodontics for dentinal decontamination before final cementation of crowns requires high-level laser therapy [24], this study investigated the antibacterial effect of this therapy at a minimum power of the used diode laser. In the present study, we used a SiroBlue diode laser at a wavelength of 660 nm. This chosen wavelength is specific to the photobiomodulation (PBM) protocol of this laser. Its applicability in tissue regeneration does not imply the use of a photosensitizer. The biomodulatory effect of diode lasers with different wavelengths was demonstrated [25,26]. A single laser-irradiation was performed, similarly to the process reported by another research [25].

Using tissue regeneration laser settings, as well as the addition of methylene blue as a photosensitizer and its washing with Kaqun water, represents an atypical protocol of PDT. Other studies have shown the effectiveness of various standard PDT protocols on both aerobic and anaerobic bacterial species [27,28,29,30]. The selection of the photosensitizer (MB) and laser power (100 mW) was based on previous works that demonstrated their efficacy [4,21,30]. MB had also a higher sterilizing effect [20] when compared with hematoporphyrin monomethyl ether (HMME). Post-PDT SEM images revealed a significant bacterial reduction; our results are in consensus with the antibacterial effect reported by other studies [4,6,20,21,31,32].

Moreover, this study evaluated the impact that photodynamic therapy had on the immediate adhesion of all-ceramic restorations. To our knowledge, no previous studies have evaluated the influence of PDT on veneers, inlays, and crowns adhesion to prepared coronary enamel and dentin, separately or together with the antimicrobial effect. Furthermore, e.max restorations are made from lithium disilicate ceramic, which provides excellent aesthetics and superior quality and strength. IPS e.max Press ceramic material contains a glass matrix, and 70% incorporated lithium disilicate crystals. IPS e.max PRESS is available as ingots with different shades and translucencies [33]. This material is used for veneers, inlays, and single crowns, as it is able to ensure translucency and resistance, with indications in both anterior and posterior teeth. The fracture resistance of lithium disilicate prosthesis is higher than that of zirconia or metal–ceramic prosthesis [34].

The longevity of indirect prosthetic restorations depends largely on the quality of the adhesion between the restorative material and the dental substrate. The veneer–cement-adhesive–tooth (enamel) interface revealed a homogeneous and uniformly thick layer of Variolink Esthetic DC adhesive cement. At the boundary between the veneer and the adhesive cement, there were areas with microcracks due to the contraction of the cement during light curing (Figure 3b), but the contact was intimate, without gaps, without areas of separation of the two materials, emphasizing a tight adhesion of cement to ceramics [35]. The evaluation of the junction between the adhesive cement and the tooth (enamel) showed very good adhesion, an adhesive system of uniform thickness, and a weakly represented but present hybrid layer (Figure 3c). No cracks, gaps, or discontinuities were identified at this level, indicating a good quality of adhesion between the adhesive cement and the tooth enamel. Our findings are similar to those reported by other studies that evaluated the adhesion of lithium disilicate ceramic bonded with the same cement that we used [36]. SEM images of tooth dentin–ceramic inlay (Figure 3d,e,f) and tooth dentin–ceramic crown (Figure 3g,h,i) highlighted a close adaptation of the prosthesis to tooth structures at different magnification levels. It is also worth mentioning that for heat-pressed lithium disilicate crowns and inlays, a marginal gap no higher than 100 µm is clinically acceptable [37,38].

In this study, we used ceramic prosthesis realized together with an occlusal or incisal bar parallel with their path of insertion, which served for assembling the universal testing machine and performing a mechanical pull-out test [39]. For every investigated group (crowns, veneers, and inlays) with and without PDT, testing was performed. Our results demonstrated that the antimicrobial protocol performed before adhesion did not statistically influence the examined parameters (load at maximum load, tensile strength, and load at upper yield). Noticeably, the obtained values were higher for every group when PDT was used (Table 1)—except for the tensile strength of veneers, which was approximatively the same with and without PDT. To our knowledge, no previous study has compared the tensile strength of cemented e.max ceramic prosthetic restorations before and after PDT using a pull-out retention test. All specimens from crown and veneer groups failed by fracture, while the inlay group failed by decementation. This is probably due to the difference in thickness between restorations—the inlays are thicker, in accordance with the cavity preparation. In our findings, load at maximum load for all the studied groups was similar when compared to the failure load reported by one study that used an unspecified luting composite for cementation [34]. Another study [40] reported higher values of the Load at maximum load than our results. This difference may appear due to the different protocol—the cementation was performed using adhesive cement different from ours, the occlusal surface was prepared flat, the wax patterns were designed digitally and milled, and because the internal surfaces of the restorations were etched with 9.5% hydrofluoric acid. A study with similar results [41] of the load at maximum load evaluated the retention of lithium disilicate crowns using bioactive cements. We did not find studies that evaluated tensile strength and load at upper yield for the types of restorations that we evaluated by pull-out test.

After assessing the results for every group of specimens, the authors evaluated the differences and similarities between them. Based on the results, we did not find any difference between veneer and crown groups when analyzing tensile strength, load at maximum load, and load at upper yield. A statistically significant difference was detected between them and the inlay group regarding load at maximum load (*p* = 0.003) and load at upper yield (*p* = 0.027). This difference may be due to the increased thickness of the inlay, compared with the other two preparations.

All specimens were submitted to SEM examination to investigate fracture areas on both tooth and ceramic surfaces. The microscopy images reveal dentin areas partially covered with the residual adhesive cement at the fracture zone of the ceramic prosthesis. The appearance of the fracture lines revealed small fragments of ceramic material above the cracked dental tissue. The adhesive cement covered the dental areas where the fracture occurred at the ceramic–cement interface and was absent where the bond had failed at the cement–tooth interface, similar to what other studies have reported [42,43].

The limitations of the present study are represented by the dehydration of samples during SEM examination. Additionally, observation of small sections may not fully reflect the characteristics of the whole sample under investigation. Concerning mechanical testing, the practitioner’s skills in performing all cementation steps can influence mechanical results. Moreover, using a single bacterial species led to a complete lack of results of the evaluation under the conditions of a biofilm.

In our future research, we intend to use complex, multispecies biofilms. In this way, by collecting oral dental plaque for contamination, in vivo conditions and the structure of a biofilm can be simulated [44,45].

Within the limitations of the in vitro conditions, the present study demonstrates the effectiveness of photodynamic therapy when it is used as a part of the cementation protocol. Additionally, it opens the way for future research to increase the applicability and use of PDT in fixed prosthodontics from an antibacterial and adhesion point of view.

## 5. Conclusions

*S. mutans* adhesion on prepared teeth and development of the bacterial biofilm was highlighted by pre-irradiation microscopic examinations.

SEM images after PDT showed a bacterial reduction, demonstrating that 100 mW laser power was sufficient to decontaminate the prepared surfaces. PDT can be considered an appropriate disinfection protocol for coronary dental tissues before the restorative phase.

Evaluation of the adhesive cementation of lithium disilicate ceramic veneers, crowns, and inlays showed a good marginal adaptation. A mechanical pull-out test revealed improved adhesion for all examined groups when PDT was applied before cementation., However, the inlay group had better bond strength when compared with the veneer and crown groups.

More studies are needed to evaluate the effectiveness of different photosensitizers and lasers on disinfection and adhesion, to extend the use of lasers in dental prosthetics.

## Figures and Tables

**Table 1:** Adhesion pull-out test.

Type of Prosthesis		Load at Maximum Load (N)	Tensile Strength (MPa)	Load at Upper Yield (N)
Crown	With PDT	265.82 ± 34.6781 ^a^	84.6 ± 8.52369 ^c^	209 ± 42.7055 ^d^
	Without PDT	257.22 ± 51.8807 ^a^	64.7 ± 12.7614 ^c^	188 ± 55.1160 ^d^
Veneer	With PDT	248.43 ± 20.9848 ^a^	79.079 ± 7.64638 ^c^	248.43 ± 35.0042 ^d^
	Without PDT	239.55 ± 35.7009 ^a^	80.571 ± 10.3334 ^c^	228.88 ± 24.2239 ^d^
Inlay	With PDT	305.98 ± 41.05021 ^b^	97.396 ± 11.03891 ^c^	290.83 ± 28.9973 ^e^
	Without PDT	302.11 ± 84.5941 ^b^	90.557 ± 15.2840 ^c^	290.05 ± 20.9471 ^e^
*p* value		0.00316	0.2135	0.02715

## Data Availability

The data reported in the present study are available on request from the corresponding author.
